# Seven genome sequences of bacterial, environmental isolates from Pony Lake, Antarctica

**DOI:** 10.1128/mra.00744-23

**Published:** 2023-12-06

**Authors:** Christine M. Foreman, Heidi J. Smith, Markus Dieser

**Affiliations:** 1 Center for Biofilm Engineering, Montana State University, Bozeman, Montana, USA; 2 Department of Chemical and Biological Engineering, Montana State University, Bozeman, Montana, USA; 3 Department of Microbiology and Cell Biology, Montana State University, Bozeman, Montana, USA; Rochester Institute of Technology, Rochester, New York, USA

**Keywords:** Antarctica, DOM

## Abstract

Dissolved organic matter (DOM) in Antarctic inland waters is unique in that its precursor molecules are microbially derived and lack the chemical signature of higher plants. Here, we report the genomic sequences of seven environmental, bacterial isolates from Pony Lake, Antarctica, to explore the genetic potential linked to DOM processing.

## ANNOUNCEMENT

The chemical composition of dissolved organic matter (DOM) depends on its precursor materials. DOM in Antarctic inland waters is unique as it is derived entirely from microbes (1 – 3).

Pony Lake (77°33′S, 166°00′E), Antarctica, is a small, brackish pond. Except for a few weeks mid-summer, the pond is frozen (4). Pony Lake has a truncated food web, with DOM produced by phytoplankton and cyanobacterial mats and transformed by heterotrophic bacteria (5, 6). Because of its unique composition, it is an International Humic Substance Society (7) DOM reference standard. We have used both cultivation-dependent and independent approaches to assess the microbial diversity and metabolic pathways (4, 8) governing the chemical composition (7, 9, 10) of DOM from Pony Lake. Genome sequences presented herein are intended to aid identification of genome features involved in the uptake and metabolism of DOM metabolites.

Sample collection, culturing, and bacterial isolation are described elsewhere (8). The cetyltrimethylammonium bromide procedure was used for extracting DNA from bacterial isolates grown to late exponential phase (11). Standard quality shotgun libraries for bacterial strains PL11, PL12, PL15, PL16, and PL17 were sequenced on the Illumina NovaSeq S4 platform (12). Libraries were prepared on the PerkinElmer Sciclone NGS robotic liquid handling workstation using the Kapa Biosystems library kit. DNA was sheared to 459 bp using a Covaris LE220 focused-ultrasonicator. DNA fragments were size selected by double-SPRI. Fragments were end-repaired, A-tailed, and ligated with Illumina compatible sequencing adaptors. Raw Illumina sequences were quality filtered using BBTools (13) per SOP 1,061 and assembled with SPAdes (≥ version v3.14.1; –phred-offset 33 –cov-cutoff auto -t 16 m 64 –careful -k 25,55,95) (14). Contigs with a length <1 kb (BBTools reformat.sh: minlength = 1,000 ow = t) were discarded. For improved-high-quality draft genomes, Pacbio SMRTbell libraries for bacterial strains PL19 and PL20 were sequenced on Pacific Biosciences (PacBio) Sequel platform (15). DNA was sheared around 10 kb using Megaruptor 3 (Diagenode) or g-TUBE (Covaris). Sheared DNA was treated with exonuclease, DNA repair enzyme mix, end-repair/A-tailing mix, and ligated with barcoded overhang adapters using SMRTbell Express Template Prep Kit 2.0 (PacBio). Libraries were purified with AMPure PB Beads (PacBio) and bound to Sequel II polymerase 2.0 using the Sequel II Binding kit 2.0. Libraries were sequenced using tbd-sample-dependent sequencing primers, 8M v1 SMRT cells, and Version 2.0 sequencing chemistry with 1 × 900 sequencing movie run times. For isolate PL19, sequences reads >5 kb were assembled with Hierarchical Genome Assembly Process [HGAP; smrtlink/8.0.0.80529, HGAP 4 (1.0)] using default settings (16). For isolate PL20, sequence reads were assembled with Flye 2.8.1 [–asm-coverage 100 –plasmid –genome-size 5,000,000 -t 64 –pacbio-raw (fasta list)] (17). Contigs with a probability of being a plasmid were identified using TensorFlow (18). CRISPR elements were identified using the programs CRISPR Recognition Tool (CRT) (19) and PILER-CR (20). All genomes were annotated in the Integrated Microbial Genomes (IMG) database (≥IMG Annotation Pipeline v.5.0.20) (21). Genome completeness was estimated using checkM (22). Bacterial taxonomic affinities were assessed by blasting the genome-sequence-acquired 16S ribosomal RNA gene using the Silva SSU database (23). Sequence details are given in Table 1.

**TABLE 1:** Summary of genome characteristics^*a*^

Platform	Organism [% match to genus]	Coverage (×)	# Contigs	Contig N/L50	Size (bp)	GC (%)	# Genes	# C	# P
Illumina	*Flavobacterium* sp. PL11 [96.5]	392.0	78	12/104,482	3,821,331	32.1	3,391	2	–
Illumina	*Flavobacterium* sp. PL12 [99.0]	458.0	65	12/110,213	3,432,272	32.63	3,075	–	–
Illumina	*Psychrobacter* sp. PL15 [99.7]	500.6	37	5/251,740	3,082,892	42.92	2,570	1	–
Illumina	*Arthrobacter* sp. PL16 [99.6]	404.9	25	3/472,694	3,689,476	66.56	3,491	–	–
Illumina	*Serratia* sp. PL17 [99.7]	143.3	27	3/533,798	5,576,906	53.42	5,488	1	–
PacBio	*Psychrobacter* sp. PL19 [99.6]	309.5	1	1/3,119,997	3,119,997	42.99	2,569	1	–
PacBio	*Sphingomonas* sp. PL20 [99.9]	200.6	5	1/4,631,974	4,826,632	64.39	4,637	–	4

^*a*^ C = CRISPR and P = Plasmid.

## Data Availability

Sequence read archive data are available through the NCBI server. Accession numbers are listed in descending order for organisms presented in Table 1: PRJNA1008773, PRJNA1008774, PRJNA1008775, PRJNA1008776, PRJNA695734, PRJNA651894, and PRJNA651895.

## References

[1] McKnight DM , Aiken GR , Smith RL . 1991. Aquatic fulvic acids in microbially based ecosystems: results from two desert lakes in Antarctica. Limnol Oceanogr 36:998–1006. doi:10.4319/lo.1991.36.5.0998

[2] McKnight DM , Andrews ED , Spaulding SA , Aiken GR . 1994. Aquatic fulvic acids in algal‐rich Antarctic ponds. Limnol Oceanogr 39:1972–1979. doi:10.4319/lo.1994.39.8.1972

[3] Foreman CM , Cory RM , Morris CE , SanClements MD , Smith HJ , Lisle JT , Miller PL , Chin YP , McKnight DM . 2013. Microbial growth under humic-free conditions in a supraglacial stream system on the cotton glacier, Antarctica. Environ Res Lett 8:035022. doi:10.1088/1748-9326/8/3/035022

[4] Foreman CM , Dieser M , Greenwood M , Cory RM , Laybourn-Parry J , Lisle JT , Jaros C , Miller PL , Chin YP , McKnight DM . 2011. When a habitat freezes solid: microorganisms over-winter within the ice column of a coastal Antarctic lake. FEMS Microbiol Ecol 76:401–412. doi:10.1111/j.1574-6941.2011.01061.x 21276026

[5] McKnight DM , Andrews ED , Spaulding SA , Aiken GR . 1994. Aquatic fulvic acids in algal-rich Antarctic ponds. Limnology & Oceanography 39:1972–1979. doi:10.4319/lo.1994.39.8.1972

[6] Brown A , McKnight DM , Chin Y-P , Roberts EC , Uhle M . 2004. Chemical characterization of dissolved organic material in pony lake, a saline coastal pond in Antarctica. Mar Chem 89:327–337. doi:10.1016/j.marchem.2004.02.016

[7] Cawley KM , McKnight DM , Miller P , Cory R , Fimmen RL , Guerard J , Dieser M , Jaros C , Chin Y-P , Foreman C . 2013. Characterization of fulvic acid fractions of dissolved organic matter during ice-out in a hyper-eutrophic, coastal pond in Antarctica. Environ Res Lett 8:045015. doi:10.1088/1748-9326/8/4/045015

[8] Dieser M , Foreman CM , Jaros C , Lisle JT , Greenwood M , Laybourn-Parry J , Miller PL , Chin YP , Mcknight DM . 2013. Physicochemical and biological dynamics in a coastal Antarctic lake as it transitions from frozen to open water. Antarct Sci 25:663–675. doi:10.1017/S0954102013000102

[9] D’Andrilli J , Foreman CM , Marshall AG , McKnight DM . 2013. Characterization of IHSS Pony Lake fulvic acid dissolved organic matter by electrospray Ionization fourier transform ion cyclotron resonance mass spectrometry and fluorescence spectroscopy. Org Geochem 65:19–28. doi:10.1016/j.orggeochem.2013.09.013

[10] Smith HJ , Tigges M , D’Andrilli J , Parker A , Bothner B , Foreman CM . 2018. Dynamic processing of DOM: insight from exometabolomics, fluorescence spectroscopy, and mass spectrometry. Limnol Oceanogr Lett 3:225–235. doi:10.1002/lol2.10082 30374456 PMC6203345

[11] William S , Feil H , Copeland A . 2012. Bacterial genomic DNA isolation using CTAB. Sigma 50:6876.

[12] Bennett S . 2004. Solexa ltd. Pharmacogenomics 5:433–438. doi:10.1517/14622416.5.4.433 15165179

[13] Bushnell B . BBTools software package (version 38.95). https://bbtools.jgi.doe.gov.

[14] Bankevich A , Nurk S , Antipov D , Gurevich AA , Dvorkin M , Kulikov AS , Lesin VM , Nikolenko SI , Pham S , Prjibelski AD , Pyshkin AV , Sirotkin AV , Vyahhi N , Tesler G , Alekseyev MA , Pevzner PA . 2012. SPAdes: a new genome assembly algorithm and its applications to single-cell sequencing. J Comput Biol 19:455–477. doi:10.1089/cmb.2012.0021 22506599 PMC3342519

[15] Eid J , Fehr A , Gray J , Luong K , Lyle J , Otto G , Peluso P , Rank D , Baybayan P , Bettman B , et al. . 2009. Real-time DNA sequencing from single polymerase molecules. Science 323:133–138. doi:10.1126/science.1162986 19023044

[16] Chin C-S , Alexander DH , Marks P , Klammer AA , Drake J , Heiner C , Clum A , Copeland A , Huddleston J , Eichler EE , Turner SW , Korlach J . 2013. Nonhybrid, finished microbial genome assemblies from long-read SMRT sequencing data. Nat Methods 10:563–569. doi:10.1038/nmeth.2474 23644548

[17] Kolmogorov M , Yuan J , Lin Y , Pevzner PA . 2019. Assembly of long, error-prone reads using repeat graphs. Nat Biotechnol 37:540–546. doi:10.1038/s41587-019-0072-8 30936562

[18] Rampasek L , Goldenberg A . 2016. TensorFlow: biology’s gateway to deep learning Cell Syst 2:12–14. doi:10.1016/j.cels.2016.01.009 27136685

[19] Bland C , Ramsey TL , Sabree F , Lowe M , Brown K , Kyrpides NC , Hugenholtz P . 2007. CRISPR recognition tool (CRT): a tool for automatic detection of clustered regularly interspaced palindromic repeats. BMC Bioinformatics 8:1–8. doi:10.1186/1471-2105-8-209 17577412 PMC1924867

[20] Edgar RC . 2007. PILER-CR: fast and accurate identification of CRISPR repeats. BMC Bioinformatics 8:1–6. doi:10.1186/1471-2105-8-18 17239253 PMC1790904

[21] Chen I-MA , Chu K , Palaniappan K , Pillay M , Ratner A , Huang J , Huntemann M , Varghese N , White JR , Seshadri R , Smirnova T , Kirton E , Jungbluth SP , Woyke T , Eloe-Fadrosh EA , Ivanova NN , Kyrpides NC . 2019. IMG/M v. 5.0: an integrated data management and comparative analysis system for microbial genomes and microbiomes. Nucleic Acids Res. 47:D666–D677. doi:10.1093/nar/gky901 30289528 PMC6323987

[22] Parks DH , Imelfort M , Skennerton CT , Hugenholtz P , Tyson GW . 2015. CheckM: assessing the quality of microbial genomes recovered from isolates, single cells, and metagenomes. Genome Res. 25:1043–1055. doi:10.1101/gr.186072.114 25977477 PMC4484387

[23] Quast C , Pruesse E , Yilmaz P , Gerken J , Schweer T , Yarza P , Peplies J , Glöckner FO . 2013. The SILVA ribosomal RNA gene database project: improved data processing and web-based tools. Nucleic Acids Res. 41:D590–D596. doi:10.1093/nar/gks1219 23193283 PMC3531112

